# Ethylene and Abscisic Acid Signaling Pathways Differentially Influence Tomato Resistance to Combined Powdery Mildew and Salt Stress

**DOI:** 10.3389/fpls.2016.02009

**Published:** 2017-01-09

**Authors:** Christos Kissoudis, Alireza Seifi, Zhe Yan, A. T. M. Tanjimul Islam, Hanneke van der Schoot, Clemens C. M. van de Wiel, Richard G. F. Visser, C. G. van der Linden, Yuling Bai

**Affiliations:** ^1^Plant Breeding, Wageningen University & ResearchWageningen, Netherlands; ^2^Biotechnology and Plant Breeding Department, Faculty of Agriculture, Ferdowsi University of MashhadMashhad, Iran

**Keywords:** abscisic acid, senescence, cell death, callose, ROS burst, chitinase

## Abstract

There is currently limited knowledge on the role of hormones in plants responses to combinations of abiotic and pathogen stress factors. This study focused on the response of tomato near-isogenic lines (NILs) that carry the *Ol-1, ol-2*, and *Ol-4* loci, conferring resistance to tomato powdery mildew (PM) caused by *Oidium neolycopersici*, to combined PM and salt stress. These NILs were crossed with the *notabilis* (ABA-deficient), *defenceless1* (JA-deficient), and *epinastic* (ET overproducer) tomato mutants to investigate possible roles of hormone signaling in response to combined stresses. In the NILs, marker genes for hormonal pathways showed differential expression patterns upon PM infection. The *epinastic* mutation resulted in breakdown of resistance in NIL-Ol-1 and NIL-ol-2. This was accompanied by reduced callose deposition, and was more pronounced under combined salt stress. The *notabilis* mutation resulted in H_2_O_2_ overproduction and reduced susceptibility to PM in NIL-Ol-1 under combined stress, but lead to higher plant growth reduction under salinity and combined stress. Resistance in NIL-ol-2 was compromised by the *notabilis* mutation, which was potentially caused by reduction of callose deposition. Under combined stress the compromised resistance in NIL-ol-2 was restored. PM resistance in NIL-Ol-4 remained robust across all mutant and treatment combinations. Hormone signaling is critical to the response to combined stress and PM, in terms of resistance and plant fitness. ABA appears to be at the crossroads of disease susceptibility/senescence and plant performance under combined stress These gained insights can aid in narrowing down targets for improving crop performance under stress combinations.

## Introduction

Plant hormones are central to plant adaptation to changing environmental conditions as well as to interactions with pathogenic and non-pathogenic organisms. To maximize fitness under different stress scenarios, resource allocation must be precisely prioritized, and to achieve that, hormonal signaling pathways are delicately interconnected and inter-regulated (Denance et al., 2013). Understanding the underlying regulatory mechanisms of hormone crosstalk is increasingly important in view of the current global climate change, which is projected to further intensify unfavorable conditions for crop production (Lobell et al., 2011; Challinor et al., 2014; Trnka et al., 2014). A significant consequence of climate change is the increased frequency of stress combinations that plants are exposed to, especially of abiotic factors in combination with pathogenic microorganisms (Garrett et al., 2006; Kissoudis et al., 2014; Suzuki et al., 2014).

Significant progress has been made in understanding hormone cross-regulation under individual stress factors, such as abiotic stress or defence response to pathogens. Abscisic acid (ABA) is the major orchestrator of adaptation and tolerance to abiotic stress (Yoshida et al., 2014), while interplay between salicylic acid (SA), jasmonic acid (JA), and ethylene (ET) regulates resistance responses to pathogens and pests (Pieterse et al., 2012). In the model plant *Arabidopsis thaliana*, SA is the main player in responses to biotrophic pathogens, while JA and ET, antagonistically with SA, mount defence against necrotrophs (Robert-Seilaniantz et al., 2011). This distinction is rather blurry in many occasions, as interactions between hormonal pathways depend on hormone concentration and timing of induction (Koornneef et al., 2008; Pieterse et al., 2009), and species-specific responses may be distinct from those reported in *Arabidopsis*. For example, in barley activation of systemic acquired resistance is under the control of ERF and WRKY transcription factors, but not of SA (Dey et al., 2014). In tomato, however, SA enhances resistance against necrotrophic pathogens such as *Botrytis*, while increasing susceptibility against biotrophs (Achuo et al., 2004).

With regard to interaction between abiotic and biotic stresses, a mostly antagonistic interaction was observed between ABA and defence signaling across many plant species. ABA negatively interacts with both SA and JA/ET signaling, compromising local and systemic acquired resistance to pathogens (Anderson et al., 2004; Yasuda et al., 2008; Ulferts et al., 2015). Increased disease resistance in ABA-deficient mutants strengthen this notion, though in many cases the increased resistance might be due to pleiotropic effects of ABA depletion (Curvers et al., 2010; Mang et al., 2012; Sanchez-Vallet et al., 2012). On the contrary, there are also evidence for positive roles of ABA signaling especially in pre-penetration defence responses through priming for cell wall fortifications and callose deposition (Ton et al., 2009; Garcia-Andrade et al., 2011). Thus, even though the majority of the studies indicate a negative role of ABA in defence responses, this does not preclude a potential beneficial contribution in specific pathosystems or at specific stages during pathogenesis.

Recent studies suggested non-additive interactions between responses to abiotic and biotic stresses at both phenotypic and molecular levels (Prasch and Sonnewald, 2013; Rasmussen et al., 2013; Kissoudis et al., 2014). The complexity of interactions under combined abiotic and biotic stress is further emphasized by the differential regulation of a significant number of SA, JA, and ET responsive genes under abiotic stress (Walia et al., 2007; Huang et al., 2008). How the up-regulation of defence signaling pathways under combined stress affects adaptation to abiotic stress has not been established yet, although there is evidence suggesting that up-regulation of SA signaling dampens ABA-mediated responses (Kim et al., 2011).

Our research is focused on the regulation of tomato resistance responses to the combination of salt stress and powdery mildew (PM) caused by *Oidium neolycopersici*. We previously demonstrated that PM resistance was negatively affected by 100 mM NaCl (EC level of 10 dS/m) in an introgression line population segregating for partial PM resistance (Kissoudis et al., 2015). Further research indicated that salt stress has the highest impact on disease susceptibility under mild stress conditions (EC∼6 dS/m, 50 mM NaCl), while more severe salinity restored resistance. Combined stress impacted plant performance significantly more than the individual stresses, which was manifested by accelerated senescence and leaf abscission. However, this response was strongly conditioned by the type of resistance to PM, as indicated by the examination of near-isogenic lines (NILs) carrying the resistance loci *Ol-1, ol-2*, and *Ol-4* (Kissoudis et al., 2016). The dominant locus *Ol-1* enhances basal defence by inducing delayed cell death (DCD) in the late stages of pathogen infection (Li et al., 2007; Seifi, 2011). The recessive gene *ol-2*, which is a mutant of the tomato *MLO* gene encoding a membrane protein, mediates resistance to PM by callose deposition and cell wall fortification to stop PM at the pre-penetration stage (Bai et al., 2008). The dominant locus *Ol-4* is an NBS-LRR gene homologous to *Mi-1* (Seifi et al., 2011), and triggers a hypersensitivity response (HR) to prevent the PM colonization after formation of primary haustoria (Bai et al., 2003; Li et al., 2006). Expression analysis of selected pathway marker genes indicated a significant role of ET and JA, which were uniquely highly upregulated in the tomato susceptible genotypes under combined stress (Kissoudis et al., 2016).

Here, we evaluated the effects of three major hormonal pathways, ABA, JA, and ET on different tomato resistance mechanisms to PM conferred by different *Ol-*genes (*Ol-1, ol-2*, and *Ol-4*). Two complementary strategies were adopted in this work. First, we monitored the expression of marker genes for different hormone pathways by using NILs that carry each of the different *Ol-*genes. Then, we evaluated whether PM resistance in these NILs is compromised under single (either salt or PM) or combined stress (salt and PM) when JA, ET, and ABA pathways are perturbated, by analyzing progenies of crosses between these NILs and ABA, JA, and ET hormone mutants. Our results provide a better understanding of how major hormonal pathways can affect tomato resistance and plant performance under combined PM and salt stress.

## Materials and Methods

### Plant and Fungal Materials

The recessive *epinastic (epi)*, and *notabilis (not)* tomato mutants and their respective backgrounds AC (Ailsa Craig), and VNF8, were obtained from the Tomato Genetic Resource Center (TGRC), University of California, Davis, CA, USA. The tomato *defenseless1* (*def1*) recessive mutant was obtained from Dr. C.A. Ryan, Washington State University. The near-isogenic lines NIL-Ol-1, NIL-ol-2, and NIL-Ol-4 [in the background of *S. lycopersicum* cv Moneymaker (MM)] confer monogenic resistance to PM through different mechanisms (Bai et al., 2005). Each of the NILs was crossed with the *epi, not*, and *def1* mutants, with the exception of the NIL-Ol-4 cross with the *not* mutant. By subsequent selfing, homozygous progenies for both the *Ol*-gene and mutations were selected in the F_3_ and F_4_ generations, and were used for evaluation of response to combined PM and salt stress.

The pathogenic fungus *O. neolycopersici* originated from infected commercial tomato plants and was maintained on MM plants in a greenhouse compartment at 20 ± 3°C with 70 ± 15% relative humidity (RH).

### Selection for the Presence of *Ol*-Genes and Hormonal Mutations

Selection for homozygous *Ol*-genes was carried out by using gene-based or tightly linked molecular markers for the resistance genes (Bai et al., 2005). The primers used for genotyping were: F-TGCTCTAACAAAATCACCAAAATC and R-AAATGGTCAAACAAAGTCTATTGAG for *Ol-1*, F-ACCCTTAAGAAATAGGGCAAA and R- ACCATCATGAACCCATGTCT for *ol-2*, and: F-GAACCGGATGTGTCCTTGAC and R-TTCTCCGAGACTTTGAACAAGA for *Ol-4*.

DNA isolation was carried out according to Wang et al. (1993) with some modifications. About 10 mg of leaf tissue was homogenized in 20 μl of 0.5 N NaOH for 5 min. Then 20 μl of 100 mM Tris-HCl was added and thoroughly mixed, and 5 μl of this homogenate was diluted with 95 μl of 100 mM Tris-HCl. The PCR reaction mix contained 0.12 μl Phire Hot Start II DNA Polymerase (Thermo Scientific), 2 μl forward primer (5 μM), 2 μl reverse primer (5 μM), 1 μl of the diluted leaf homogenate as a DNA template and 1 μl of PVP (10% w/v) as a chelating agent for impurities, into a final volume of 11 μl. The amplification profile was 40 cycles of 98°C for 5 s, 54°C for 5 s, and 72°C for 10 s.

Different selection approaches were used to select homozygous plants for hormonal mutation, depending on whether the gene and the polymorphism underlying the mutation are known. The *not* mutation is well-characterized and is the consequence of a specific A/T base pair deletion in the coding sequence resulting in a frameshift mutation (Burbidge et al., 1999), indicating that it is a null mutant. Homozygous plants for the *not* mutation were selected based on sequencing a PCR fragment harboring the A/T mutation at position 597 of the ORF (primers used: *not*-F: GTTCGAAACGGAGCTAACCC, *not*-R: AACAAGTCCGAAGAGCCCA). The gene mutation causing the *epi* phenotype is not known, but mutant seedlings carrying the *epi* mutation are significantly shorter that wild ones when germinated in the dark (Barry et al., 2001). Accordingly, seeds were germinated in the dark and plants showing no etiolation were selected as homozygous plants carrying the *epi* mutation. Selection for the *def1* mutant was done based on the fact that this mutation affects JA biosynthesis (Howe et al., 1996). To test this, single leaflets of the wild type (WT), JA deficient parental lines were pierced and the induction of expression of the JA marker leucine aminopeptidase A (LapA) was monitored 24 h after wounding with qRT-PCR using primers F-ATCTCAGGTTTCCTGGTGGAAGGA, R-AGTTGCTATGGCAGAGGCAGAG. RNA isolation was performed with a MagMAX^TM^-96 Total RNA Isolation Kit in a KingFisher^TM^ Flex Magnetic Particle Processor according to manufacturer’s instructions, and expression of the LapA gene was evaluated following the method described in the gene expression section bellow. An average of 100-fold difference in expression was observed between WT plants and the homozygous *def1* mutant, indicating that *LapA* marker gene could be used safely as a qualitative marker for selecting the *def1* mutantion.

### Experimental Conditions and Treatments

Experiments were carried out in the greenhouse with a photoperiod regime of 16 h light and 8 h dark, and 70% RH. Additional lighting (100 Wm^-2^) was supplied if the incoming radiation was below 200 Wm^-2^. Plants were grown in pots filled with vermiculite irrigated with half strength Hoagland nutrient solution at regular intervals till leaching of the solution, in order to avoid accumulation of nutrients and NaCl.

The experiments were carried out twice in two different years (2013 and 2014) in the period of April–May. In the first experiment, the response of progenies of NILs × mutant crosses (named *Ol-1* × *def, ol-2* × *def, etc*.) that were homozygote for both the *Ol* gene and the mutation, or only homozygote for the *Ol* gene without the mutation, to PM was evaluated under normal and salt stress conditions For each combination, 6–8 plants were tested. Three-week-old plants were watered with half strength Hoagland solution containing either zero (no salt stress) or 50 mM NaCl (mild salt stress). Eight days after the initiation of salt stress treatments, plants were inoculated with PM by uniformly spraying a suspension of 5 × 10^4^ conidia per ml prepared by washing conidial spores from leaves of heavily infected (sporulation stage) MM plants. Plants were grown for another 20 days after inoculation.

In the second experiment, all NIL × mutant crosses were similarly assessed, excluding the crosses that carried only the *Ol*-genes and not the hormonal mutations. In addition, we included a non-PM treatment (only salt stress): half of the plants from *Ol-1* × *epi* or *Ol-1* × *not* (selected based on their explicit phenotypes) were spatially isolated 8 days after the salt treatments and were not sprayed with PM. The plants thus were exposed to all possible treatment combinations (no salt stress/not inoculated, no salt stress/inoculated, salt stress/not inoculated, salt stress/inoculated). Plants were grown for another 20 days after the inoculation.

### Plant Performance Evaluation under Salt Stress and PM

The disease severity was assessed at 10, 15, and 25 days post-inoculation (dpi) for the first experiment, and at 15 dpi for the second experiment, as disease index (DI) on a scale from 0 (no PM symptom) to 5 (heavy PM infection) as described before (Kissoudis et al., 2014). In addition to DI, a measure of senescence development [senescence index (SI)] was introduced to describe the accelerated senescence phenotypes observed at the late stages of infection under salt stress: 0 = healthy plant, 1 = 0.1–10% of foliar area affected, 2 = 10–20% area affected with yellowing and moderate wilting, 3 = 20–30% area affected with severe wilting, 4 = 30–50% area affected with severe wilting and moderate leaf abscission, and 5 = > 50% area affected with severe wilting and leaf abscission.

### Ion Content Analysis

The five youngest leaves (from the top of the plant) were sampled at 20 dpi, the endpoint of the second experiment, in order to examine differences in actively growing tissues, potentially linked to growth performance, and avoid the severely senescing bottom leaves of susceptible genotypes under combined stress conditions. The concentration of Na^+^, Cl^-^, K^+^, PO_4_^3-^, SO_4_^2-^, Mg^2+^, and Ca^2+^ was measured with ion chromatography as described previously (Kissoudis et al., 2015).

### *In situ* Histological Analyses of H_2_O_2_ Accumulation and Callose Deposition

Leaf disks (1.3 cm in diameter) were taken from leaflets of the 4th leaf counting from the bottom on the 3rd day after pathogen inoculation. To ensure uniformity, leaf disks were taken from the middle of the leaflets on both sides of the central vein. Staining was carried out in 24-well plates, where leaf disks were placed with the abaxial side up. For H_2_O_2_ visualization, leaf disks were stained in 1 mg/ml DAB (3-3′-diaminobenzidine), pH 3.7 for 16 h in the dark and were subsequently transferred to 96% ethanol for 24 h to remove chlorophyll (Martinez de Ilarduya et al., 2003). Leaf disks were mounted on glass slides with 70% glycerol.

Callose deposition visualization was performed according to the described method (Ton et al., 2005; Luna et al., 2011) with slight modifications. Leaf disks were initially placed in 96% ethanol to remove chlorophyll and after a 1-min wash in 0.07 M K_2_HPO_4_ (pH 9), were stained for 2 h in 0.02% (w/v) aniline blue in 0.07 M K_2_HPO_4_ (pH 9) at room temperature. Leaf disks were mounted on glass slides with 70% glycerol. Callose deposit was quantified from digital photographs by the number of white pixels (fluorescence, related to callose intensity) relative to the total number of pixels covering plant material using the software Adobe^®^ Photoshop^®^ CS6.

### Gene Expression Analysis and Pathogen Quantification

For the time course expression study on hormonal marker genes in the NILs challenged only with PM, the same time series as used previously for cDNA-AFLP profiling (Li et al., 2007) were used in this experiment. In brief, this time series included cDNAs from plants of MM, NIL-Ol-1, NIL-ol-2 and NIL-Ol-4, and non-inoculated (mock) and PM-inoculated leaves (2nd and 3rd) of three plants per line were collected at 1, 3, 5, 7, and 9 dpi. For each line, the cDNAs from mock samples from different time points were pooled and used as calibrator for qRT-PCR analysis.

To evaluate the expression of stress, defence, and hormone marker genes under salt and PM stresses, leaflets from the 3rd and 4th leaves (counting from the bottom) were sampled at 6 dpi, when pathogen mycelium growth was not yet visible. Sampling for pathogen quantification was carried out at 14 dpi, when pathogen growth from the primary infection had reached its peak using leaflets from the 4th and 5th leaf counting from the bottom. For each genotype, 4–5 plants were used.

The RNA for gene expression analyses was isolated with the RNeasy Plant Mini Kit (Qiagen). The isolated RNA was treated with DNase I (Invitrogen) to remove residual DNA. cDNA synthesis was performed using 1 μg RNA template by iScript^TM^ cDNA Synthesis Kit (BioRAD). qRT-PCR was conducted using the iQ SYBR Green supermix (Bio-Rad) and the CFX96 Real-Time system (Bio-Rad). The reaction mix contained 5 μl 2× iQ SYBR GREEN super mix, 1 μl forward primer (3 μM), 1 μl reverse primer (3 μM), and 3 μl cDNA (or DNA, 20 ng) template, in a final volume of 10 μl. Thermocycling condition was 95°C for 3 min, followed by 40 cycles of 95°C for 30 s and 60°C for 30 s. Primers for tomato elongation factor 1α (EF) were Fw-EF-GGAACTTGAGAAGGAGCCTAAG and Rv-EF-CAACACCAACAGCAACAGTCT (Gao et al., 2014). The primers used to monitor the expression of tomato genes are described in **Supplementary Table S1**. Relative expression was calculated using the 2^-ΔΔCt^ method (Livak and Schmittgen, 2001). Plant and fungal DNA for pathogen quantification analysis was extracted with DNeasy Plant Mini Kit (Qiagen). Primers used for fungal quantification were Fw-On-CGCCAAAGACCTAACCAAAA and Rv-On-AGCCAAGAGATCCGTTGTTG.

## Results

### Expression Pattern of Marker Genes for Hormonal Pathways in NILs

**FIGURE 1:**
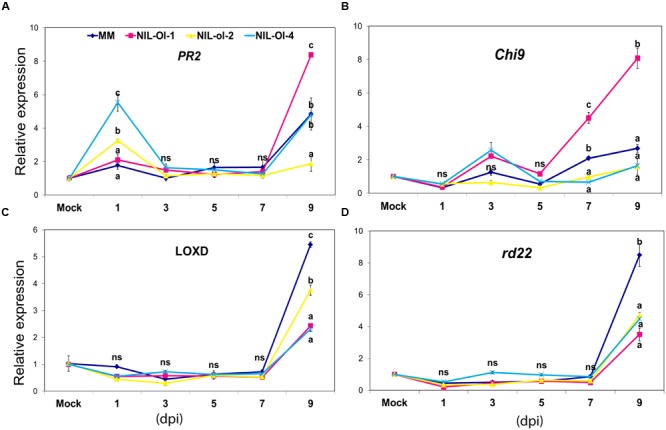
**Expression of (A)** *PR-2*, **(B)** rd22, **(C)** *Chitinase9*, and **(D)** *LOXD* (markers for SA, ABA, ET and JA and pathways, respectively) in MM, and NIL-Ol-1, -ol-2, and -Ol-4 in a time-course after inoculation with PM. Second and third leaves were sampled at 1, 3, 5, 7, and 9 days post-inoculation (dpi) from powdery mildew (PM)-inoculated and -non-inoculated (mock) plants. Statistically significant differences (*P* ≤ 0.05) between genotypes (within each timepoint of measurement) are designated with different letters. Error bars represent standard error of mean, (*n* = 3); ns, non-significant:

To monitor changes in JA, SA, ET, and ABA pathways, the expression level of marker genes for these pathways were measured in the NILs and MM, in a time course from 1 to 9 days after inoculation with PM. Significant differences were observed in the expression patterns and in the magnitude of induction for some of these marker genes in the NILs and MM (**Figure 1**).

Salicylic acid induces expression of a group of pathogenesis-related genes (*PR* genes) in *Arabidopsis* including *PR-2*, which is often used as a marker gene for the SA pathway (Uknes et al., 1992). The tomato *PR-2* gene (Domingo et al., 1994) was induced in response to *Phytophthora infestans* as well as in response to benzothiadiazole (BTH, an analog of SA; Beyer et al., 2001). Therefore, we used *PR-2* as a marker gene for the SA pathway in this study (see *SlPR-2* in **Supplementary Table S1**). At 1 dpi, there was an induction in the *PR-2* expression in NIL-ol-2 and NIL-Ol-4, but very little induction in NIL-Ol-1 and MM. At the last time point (9 dpi), the *PR-2* expression was increased in MM, NIL-Ol-1, and NIL-Ol-4, with the highest level in NIL-Ol-1 (approximately ninefold induction compared to non-inoculated plants).

The ET pathway signaling was monitored through the expression of the *Chitinase9* (*Chi9*) gene, which has been used as a marker gene for ET pathway in tomato (Barry et al., 2001). The expression level of *Chi9* did not show great fluctuations across genotypes and time points with the exception of NIL-Ol-1, in which a marked up-regulation was observed in the later time points (4.5- and 8-fold induction at 7 and 9 dpi, respectively).

The *lipoxygenase D (LOXD*) gene has been shown to be induced by JA in tomato (Heitz et al., 1997), thus we used this gene as a marker for the JA pathway. Its expression was relatively stable or slightly down-regulated across all genotypes till 7 dpi, but a marked up-regulation was observed in all genotypes at 9 dpi, which was strongest in MM and NIL-ol-2 (increase of approximately six and fourfold, respectively, compared to control conditions).

The *Arabidopsis rd22* gene is ABA-responsive (Shinozaki et al., 2003). By performing TBLASTN in tomato Unigene database^1^ a homologue of this gene in tomato (EU679376.1) was retrieved and used as the tomato *rd22* orthologue. Similar to the JA marker *LOXD, rd22* expression was relatively stable among genotypes in 1–7 dpi, and was significantly up-regulated at 9 dpi. MM showed the highest expression, 8.5-fold increase compared to control conditions, while all NILs exhibited a fourfold upregulation.

### Effects of Hormonal Mutants on the PM Resistance in the NILs under Combined Stresses

The mutants and their background lines, as well as their progeny that were homozygous for individual *Ol-*genes but do not carry the hormone mutations (null segregants) were evaluated for PM susceptibility. The hormone mutants and their background lines were all susceptible to PM, with a susceptibility level similar to that in MM. The null segregants, however, were as resistant as the NILs, suggesting that the resistance conferred by the *Ol*-genes was not affected by the genetic background in the crosses (data not shown).

**FIGURE 2:**
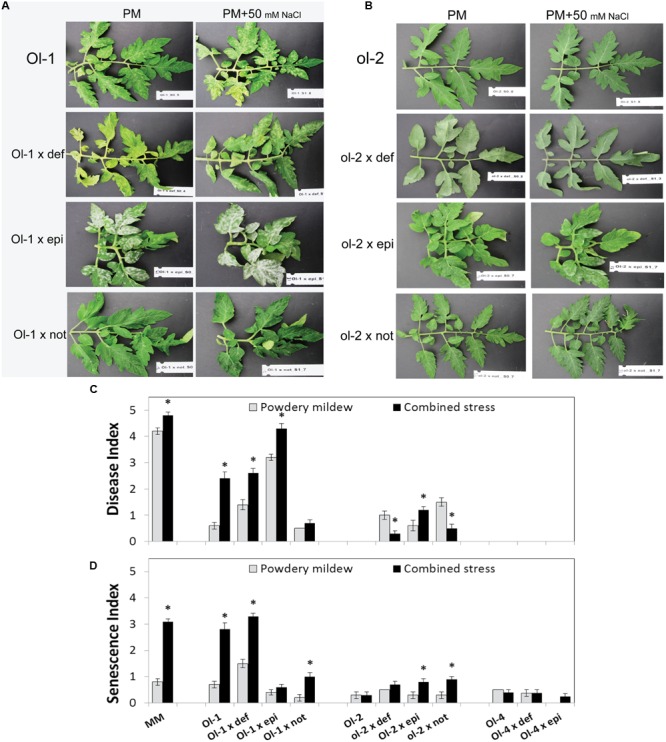
**Leaf phenotypes of (A)** NIL-Ol-1 and **(B)** NIL-ol-2 and their respective mutants under PM and combined stress. **(C)** Disease and **(D)** senescence index of NIL-O-1, -ol-2, -Ol-4, the recurrent parent MM and their crosses with different hormone mutants under PM individually (no salt stress) and in combination with 50 mM NaCl measured at 15 dpi. Error bars depict standard error (*n* = 6). *Ep*i, *epinastic* (ET overproducer); *not, notabilis* (ABA deficient); *def, defenseless1* (JA deficient). Asterisks denote statistically significant pairwise differences (*P* ≤ 0.05) between PM and combined stress (PM with salt) treatments for each genotype. Error bars represent standard error of mean.

Similar to our previous results, mild salt stress significantly increased the PM susceptibility of MM and NIL-Ol-1, while the resistance level of NIL-ol-2 and NIL-Ol-4 was not affected. When combined with hormone mutants, the resistance conferred by *Ol-1* and *ol-2* was significantly affected, but the resistance conferred *Ol-4* was not (**Figures 2A–D**).

Resistance conferred by the *Ol-1* gene was compromised in plants carrying the *epi* mutation (e.g., average DI of 3.2 for *Ol-1 × epi* compared to 0.6 for NIL-Ol-1), with susceptibility increasing even further under salt stress (DI of 4.3 with salt combination compared to 3.2 without salt, **Figure 2C, Supplementary Table S2**). The significant increase in susceptibility of the *Ol-1 × epi* plants was accompanied by almost complete abolishment of the accelerated senescence and cell death symptoms observed in NIL-Ol-1 under salt stress (SI = 0.6 for *Ol-1 × epi* compared to SI = 2.8 for NIL-Ol-1, **Figure 2D**). The *Ol-1 × not* plants showed a level of resistance similar to NIL-Ol-1 plants without salt stress. However, ABA deficiency markedly suppressed the susceptibility response of *Ol-1* under salt stress (DI = 0.7 for *Ol-1 × not* compared to DI = 2.4 for NIL-Ol-1 under combined stress, **Figure 2C**), and additionally reduced the accelerated senescence and leaf abscission phenotype (SI = 1 for *Ol-1 × not* compared to SI = 2.8 for NIL-Ol-1, **Figure 2D**). JA deficiency impacted senescence in PM treated *Ol-1 × def* plants with increased yellowing and abscission of older leaves (**Supplementary Figure S1**).

For the *ol-2-*mediated resistance, increased susceptibility was observed in o*l-2 × def, ol-2 × epi*, and *ol-2 × not* plants. Under salt stress, this susceptibility was significantly further increased for *ol-2 × epi* plants (DI = 1.2 with salt compared to DI = 0.8 without salt), while it was significantly decreased for *ol-2 × def* and *ol-2 × not* plants (DI = 0.3 and 0.5 with salt compared to DI = 1 and 1.5, respectively, without salt, **Figure 2C, Supplementary Table S2**). Slightly higher senescence was observed for *ol-2 × epi* and *ol-2 × not* under combined stress in comparison to NIL-ol-2 (**Figure 2D**).

**FIGURE 3:**
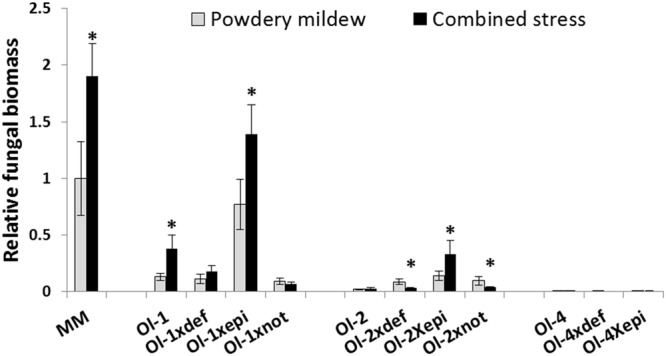
**Relative *Oidium neolycopersici* fungal biomass [calculated as the ratio of fungal ITS gene amplification in comparison with tomato EF1a and normalized with the values of MM under PM infection (no salt stress)] in MM and NIL-Ol-1, -ol-2, and -Ol-4 and their respective mutants under PM infection alone and in combination with 50 mM NaCl.** Asterisks denote statistically significant pairwise differences (*P* ≤ 0.05) between PM and combined stress (PM with salt) treatments for each genotype. Error bars represent standard error of mean (*n* = 4).

Powdery mildew quantification by qPCR was in line with the visual scoring. In many occasions even greater differences between genotypes or treatments were observed. Only for NIL-Ol-1 plants and *Ol-1 × def* plants under combined stress the qPCR results revealed a smaller difference compared to what our visual scoring suggested, potentially due to the senescence symptoms leading to an overestimation of the visual disease score (**Figure 3**).

### Performance and Fitness Cost of *epi* and *not* Mutations in NIL-Ol-1 and NIL-ol-2 under Combined Stress

Explicit phenotypes were observed for *Ol-1 × epi, Ol-1 × not, ol-2 × epi, ol-2 × not* plants. These lines were studied in more detail under control conditions, salt stress (50 mM), PM inoculation, and combined salt stress and PM inoculation, allowing an assessment of growth performance costs under the different stress conditions. These *Ol*-gene and mutant combinations are particularly interesting as ABA is the major hormone orchestrating abiotic stress responses in plants (Yoshida et al., 2014), while ET signaling was shown to be crucial for plant susceptibility and senescence responses under combined stress (Kissoudis et al., 2016).

**FIGURE 4:**
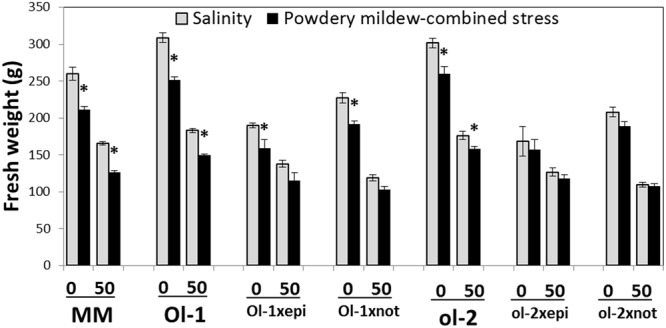
**Aboveground biomass (FW) of MM, NIL-O-1, -ol-2, and their crosses with the hormone mutants under control conditions (0) and salt stress (50 mM NaCl) on the *x*-axis, and with or without PM (black vs. light gray).** Level 0 for salinity stress corresponds to stress-free control conditions, while level 0 for PM-combined stress corresponds to PM infection alone (no salt stress). Asterisks denote statistically significant pairwise differences (*P* ≤ 0.05) between control/salinity and PM/combined stress for each genotype. Error bars represent standard error of mean (*n* = 4).

The *Ol-1 × epi* and *ol-2 × epi* plants had reduced biomass under conditions without stress compared to the corresponding NIL lines, but exhibited higher relative stress tolerance (biomass under salt stress relative to biomass under control conditions). In contrast and as expected, ABA deficiency conferred by the *not* mutation further reduced biomass under salt stress relative to the control conditions (**Figure 4**). PM resulted in a decrease in aboveground fresh weight in all *Ol*-gene × mutant combinations. The combination of salt and PM imposed an even greater growth penalty than salt stress alone. While the reduction in performance caused by PM under combined salt stress relative to salt stress alone was lower in the *not* mutant crosses, the growth reduction resulting from the salt stress *per se* was far greater in these ABA-deficient plants than in any of the other tested plants (**Figure 4**).

Ion content, and especially Na^+^ and Cl^-^ concentration was shown to impact PM susceptibility (Kissoudis et al., 2016). Both the *epi* and *not* crosses with NIL-Ol-1 and NIL-ol-2 accumulated a higher amount of Na^+^ and Cl^-^ under salt stress compared to the parental NILs (**Supplementary Figure S2**). However, the *Ol-1 × epi* and *ol-2 × epi* plants exhibited a significant reduction in the concentration of Na^+^ and Cl^-^ under combined PM and salt stress compared to salt stress only, while the crosses with *not* mutant had slightly increased Na^+^ and Cl^-^ concentrations under these conditions. K^+^ content was higher in *Ol-1 × epi* and *ol-2 × epi* plants in most of the treatments.

### Histological Analysis of Callose Deposition and H_2_O_2_ Accumulation

**FIGURE 5:**
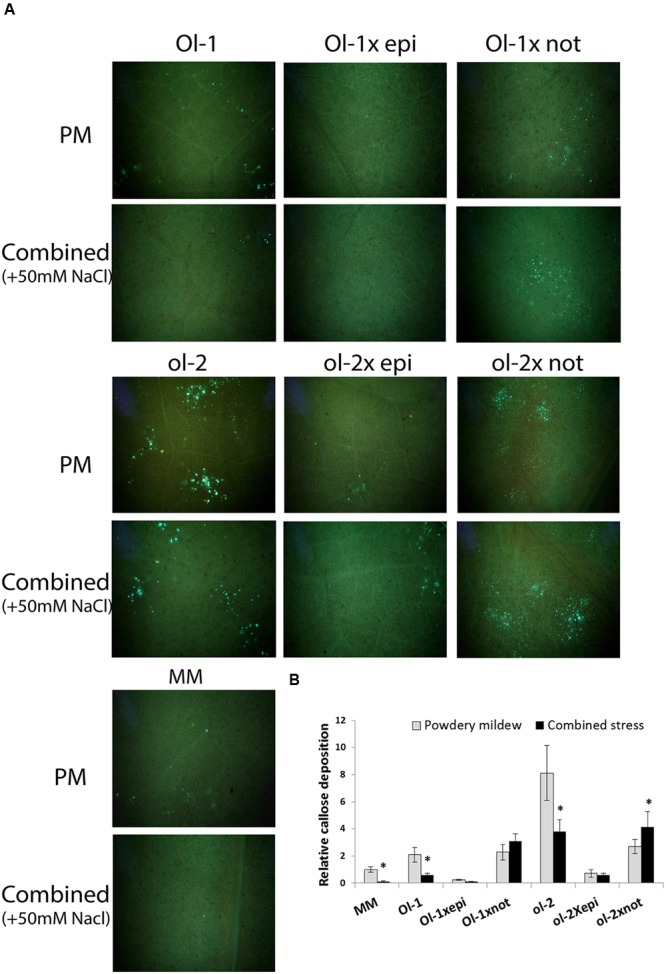
**(A)** Callose deposits in leaves of MM, NIL-Ol-1 and ol-2 and their respective crosses with *epi* and *not mutants* as visualized with UV microscopy after aniline blue staining, **(B)** quantification of callose deposition relative to MM under PM infection. Asterisks denote statistically significant pairwise differences (*P* ≤ 0.05) between PM and combined stress (PM with salt) treatments for each genotype. Error bars represent standard error of mean.

Callose deposition at the attempted sites of pathogen penetration increases plant resistance and is a major hallmark of *ol-2-*mediated resistance (Bai et al., 2008; Ellinger et al., 2013). As shown previously (Kissoudis et al., 2016), NIL-ol-2 exhibited increased callose deposits compared to NIL-Ol-1 and MM upon PM infection, and additional salt stress decreased callose deposits in all genotypes (**Figure 5**). Under only PM infection, the *epi* mutation resulted in a near complete absence of callose deposits in the crosses with *Ol-1* and *ol-2*. The *not* mutation did not significantly affect callose deposits in *Ol-1 × not* plants, but led to a threefold decrease of callose deposits in *ol-2 × not* plants (**Figure 5**, only PM infection). However, under combined stress, *Ol-1 × not* and especially *ol-2 × not* exhibited denser callose deposits compared with PM only.

**FIGURE 6:**
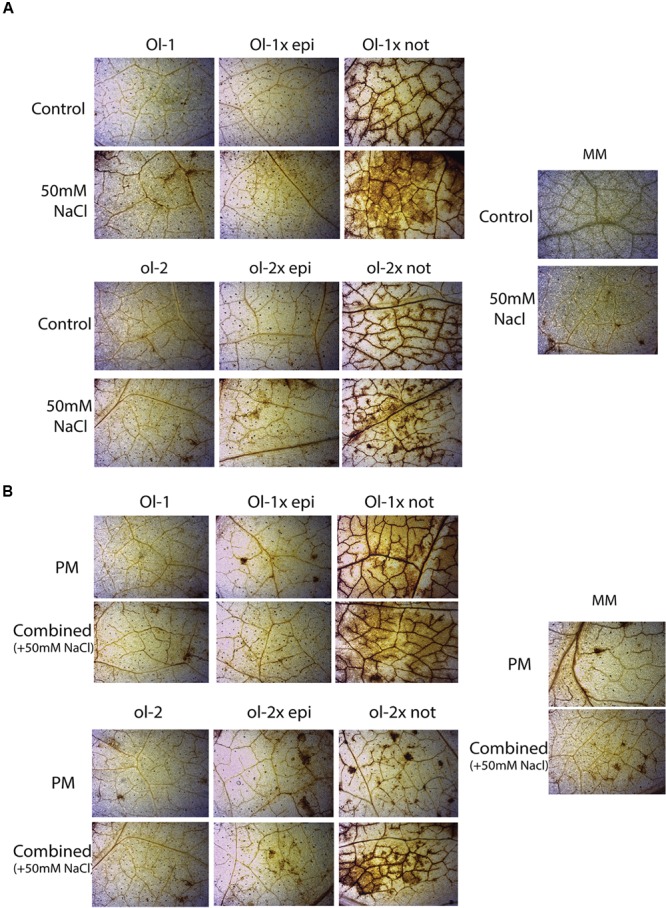
**H_2_O_2_ visualization after DAB staining in MM, NIL-Ol-1 and ol-2 and their respective crosses with *epi* and *not* mutants under (A)** control and salt stress, **(B)** PM and combined stress.

Examination of H_2_O_2_ content with DAB staining indicated slightly higher ROS production in MM, NIL-Ol-1, and NIL-ol-2 under individual stress (PM infection or salt stress) or combined stresses, compared to control plants of each genotype (**Figure 6**). The *epi* mutation did not influence H_2_O_2_ accumulation in the NILs. However, a massive H_2_O_2_ increase was observed in both *Ol-1 × not* and *ol-2 × not* plants.

### Expression Analyses

**FIGURE 7:**
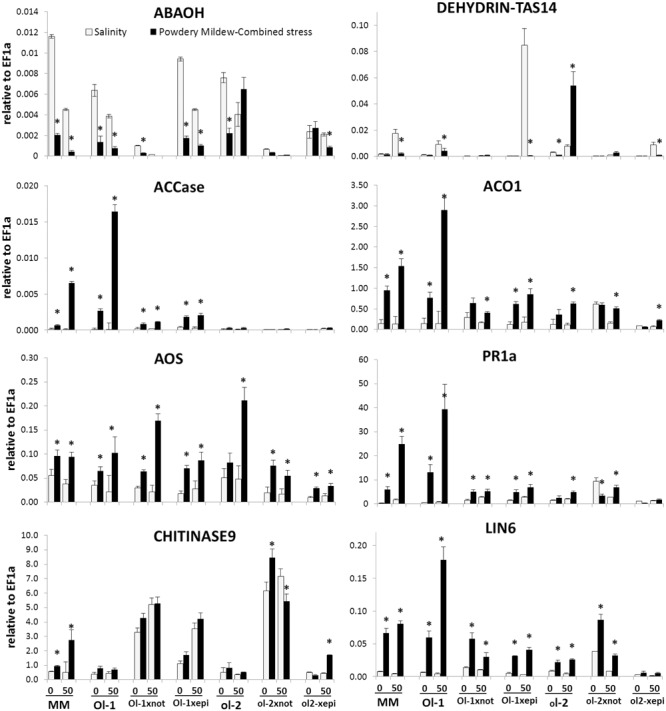
**Expression of gene-markers for hormonal, abiotic, and biotic stress signaling pathways in MM, NIL-Ol-1 and ol-2 and their respective crosses with *epi* and *not* mutants, relative to *EF1a*, which was used as a housekeeping gene.** Treatment and labeling scheme are the same as **Figure 4**. Asterisks denote statistically significant pairwise differences (*P* ≤ 0.05) between control/salinity and PM/combined stress for each genotype. Error bars represent standard error of mean (*n* = 4).

Gene expression of additional marker genes for the biosynthesis and signaling of major hormonal pathways, ROS, antioxidant, and ion homeostasis pathways involved in abiotic and biotic stress responses of tomato was monitored at 6 dpi, prior to visible PM symptoms (**Figure 7, Supplementary Figure S3**). The expression of the ABA biosynthesis gene *NCED* was either reduced (in *Ol-1 × epi* plants) or stable (in *ol-2 × epi* plants) under combined stress compared to salt stress only. In the *not* mutant this gene contains a mutation that causes a frameshift mutation. It may be transcribed but does not code for a functional enzyme. ABA deficiency in *not* is in line with the modest expression levels (significantly lower compared to NILs) of the ABA catabolic gene, *ABAOH*, and the dehydrin gene, *DHN-TAS* under all conditions. Dehydrin expression was highly induced in *Ol-1 × epi* and *o1-2 × epi* plants under salt stress (50- and 6-fold, respectively), but this response was completely abolished under combined stress, while it was exceptionally induced under combined stress in NIL-ol-2.

Under combined stress, an induction of ET biosynthetic genes *ACCase* and *ACCox (ACO1)* was observed in NIL-Ol-1, accompanying the increased PM susceptibility and senescence response. This induction was significantly reduced in *Ol-1 × epi* and *Ol-1 × not* plants. On the other hand, *CHI9* was vastly induced in both *Ol-1 × not* and *ol-2 × not* plants (up to 20-fold compared to NIL-Ol-1 and NIL-ol-2) and these expression levels were maintained under all stress treatments.

Expression levels of *AOS* and *LOXD* genes, nodes of the JA pathway, were significantly reduced in *ol-2 × epi* and *ol-2 × not* crosses under salt and combined stress treatments (reductions up to sixfold). *PR1a* induction in NIL-Ol-1 after PM and combined stress (25- and 70-fold higher, respectively, compared to non-stress conditions) was greatly reduced in both *Ol-1 × epi* and *Ol-1 × not* plants, despite the higher basal expression in these plants. The strong induction of invertase *LIN6* observed in NIL-Ol-1 under combined stress was greatly reduced in *Ol-1 × epi* and *Ol-1 × not* plants.

## Discussion

### The *epi* Mutation Compromises the *Ol-1*- and *ol-*2-Mediated PM Resistance

The *Ol-1* gene confers incomplete PM resistance by inducing DCD upon PM infection (Li et al., 2007; Seifi, 2011). The gene is not cloned yet, but it is likely a non NBS-LRR gene, enhancing basal defence. The *ol-2* gene is a *mlo-*mutant and mediates resistance to PM by callose deposition to stop PM at penetration stage (Bai et al., 2005). The PM resistance mediated by the *Ol-1* and *ol-2* genes was compromised by the *epi* mutation that is reported to produce high levels of ET (Fujino et al., 1988). This is in line with the established role of ET in susceptibility for biotrophic pathogens, in *Arabidopsis* and several other plant species, through negative interaction with SA signaling (Pieterse et al., 2009). ET signaling is also reported to be involved in disease symptom development caused by *Xanthomonas campestris* pv. *vesicatoria* in tomato (O’Donnell et al., 2003). Salt stress had an additional negative effect on the PM susceptibility of *Ol-1 × epi* and *ol-2 × epi* plants, indicating additive effects of this abiotic stress and ET in compromising *Ol-1-* and *ol-2-*mediated resistance. Salinity stress was shown to increase ET concentration in tomato (Amjad et al., 2014).

The overproduction of ET in the tomato *epi* mutant (Fujino et al., 1988) was reflected by the 10-fold induction of *ACCase* expression compared to WT (relative expression of 0.000049 in NIL-Ol-1 compared to 0.000372 in *Ol-1 × epi, p* < 0.001). No differences were observed in *ACO1* expression, the final enzyme in ET biosynthesis (relative expression of 0.140 in NIL-Ol-1 compared to 0.126 in *Ol-1 × epi, p* = 0.263). This is in accordance with the previous study that showed ACC (the product of ACCase and the rate-limiting compound for ET synthesis) is increased in the *epi* mutant (Fujino et al., 1988).

The *epi* is a mutant with pleiotropic phenotypic effects such as reduced growth and leaf epinasty (**Supplementary Figure S1**) (Fujino et al., 1988; Barry et al., 2001). There is a striking difference between the increased senescence and cell death observed in NIL-Ol-1, and the complete absence of senescence and cell death in *Ol-1 × epi* plants under combined stress, which seems to contradict the known promotion of senescence by ET (Penfold and Buchanan-Wollaston, 2014). ET overproduction is also shown to stimulate ROS production and the accompanying symptoms (Bartoli et al., 2013), but this was not observed in the *epi* plants. In NIL-Ol-1, increased susceptibility under combined stress was accompanied by an induction in the expression of ET biosynthesis and responsive genes *ACC* and *ACO1*, but not for *CHI9*. In *Ol-1 × epi* plants, this increase was only modest for *ACC* and *ACO1*, which might indicate that ET biosynthesis may not have exceeded a threshold level that would impact senescence. Similar observations on the lack of important ET-induced symptoms such as increased senescence were reported previously for this mutant (Barry et al., 2001). Additional pleiotropic alterations at the cellular level were observed for *epi* by others, especially changes in the epidermal cells which differ from the WT by having a more round-shaped and swollen cells (Barry et al., 2001). These changes can be functionally significant for biotic stress responses through a potential effect on the cytoskeleton dynamics and the secretion and deposition of anti-fungal compounds. Manipulation of these processes resulted in a significant effect on exocytosis mechanisms, which are linked to the transport of antifungal compounds at the site of infection and increased susceptibility in PAMP-mediated resistance (PTI), but did not affect HR-mediated resistance (Hardham et al., 2007; Miklis et al., 2007; Henty-Ridilla et al., 2014). This is similar to our findings of reduced callose deposition in *Ol-1 × epi* and *ol-2 × epi* plants while the HR-based resistance conferred by *Ol-4* was not affected by the *epi* mutation.

### The *not* Mutation Differentially Affects PM Resistance Mediated by the *Ol-1* and *ol-2* Genes

The *not* mutation, which induces ABA deficiency (Mulholland et al., 2003), had both positive and negative impacts on disease resistance conferred by the *Ol*-genes. It slightly, but significantly, increased susceptibility of NIL-ol-2 after PM infection, while no significant changes were observed for NIL-Ol-1. Under combined stress, the increased susceptibility and senescence of NIL-Ol-1 was significantly alleviated in the *Ol-1 × not* plants (**Figure 2**). In *ol-2 × not* plants a slight decrease of susceptibility was also observed under combined stress. These results indicate a complex interaction between ABA signaling and disease resistance in alignment with a number of other studies (Audenaert et al., 2002; De Torres Zabala et al., 2009; Curvers et al., 2010; Mang et al., 2012), while the salt stress adds another layer of complexity. Both ROS production (increased) and callose deposition (decreased) were significantly affected in *Ol-1 × not* and *ol-2 × not* plants and might underlie the differential resistance responses of these plants.

A ROS-induced oxidative burst was shown to contribute to defense against *Botrytis cinerea* in the tomato ABA-deficient mutant *sitiens* (Asselbergh et al., 2007). However, recent findings indicate only a minimal effect of the ROS-induced oxidative burst on pathogenicity (Samalova et al., 2014). In *ol-2 × not* plants, the reduced callose deposition may have facilitated PM penetration, with enhanced growth of the pathogen overriding the impact of increased ROS levels. The addition of salt stress partially decreased disease symptoms in *ol-2 × not* plants, accompanied by increased callose deposition. The increased callose deposition under combined salt and PM stress may result from the partial restoration of ABA signaling by exposure to stress (Mulholland et al., 2003), positively affecting callose deposition. The *not* mutant has about 10–15% of the ABA levels of the WT (Mulholland et al., 2003). The addition of salt stress may have resulted in induction of additional ABA responsive tomato *NCED* genes; the ABA marker *DHN-TAS* was induced 10-fold.

The elevated levels of Na^+^ and Cl^-^ concentration under combined stress might contribute to salinity-induced increased resistance, as a result of ion toxicity to the fungus. The levels observed in the *not* mutants at 50 mM NaCl were similar to the levels observed in MM plants under 150 mM NaCl, and this was shown to reduce disease progression (Kissoudis et al., 2016). Alternatively, the *not*-induced resistance may be linked to the unique increase in the expression of *CHI9* (**Figure 7**), which is considered to be a component of ET signaling in tomato (Wu and Bradford, 2003) and has direct antifungal properties (Hong and Hwang, 2006).

The most pronounced effect of the *not* mutation under combined stress was the marked attenuation of senescence and leaf abscission in *Ol-1 × not* plants. This occurred despite of very high levels of ROS, which are known to be associated with senescence (Gregersen et al., 2013), although H_2_O_2_ alone was insufficient in triggering cell death in tobacco in response to bacteria (Mur et al., 2005). Our results indicate that ABA induces senescence under combined stress, with recent studies supporting this finding (Yang et al., 2014). Uncontrolled cell death and senescence under combined stress may therefore be mediated through the ABA signaling pathway. The reduced expression of ET biosynthesis and response genes in the *not* crosses with *Ol*-genes suggests that ET signaling regulation may be involved in this phenotypic response. Both synergistic and antagonistic regulation of ABA and ET have been described, but ABA appears to enhance ET levels under abiotic stress (Albacete et al., 2009), which is in agreement with our results. Further support for a role of ET in the combined stress-induced phenotype of the *not* crosses with *Ol*-genes is given by the fact that the ET level in *not* and *sitiens* ABA deficient mutants is lower compared to WT plants (Nitsch et al., 2012).

### Hormonal Pathways Expression during PM Pathogenesis and Connections with the Phenotypes of NILs × Mutant Crosses

Ethylene signaling is induced in NIL-Ol-1 late during the time course analysis and the epinastic mutation might be disrupting this pattern resulting in increased susceptibility (**Figure 1**). Stress-induced ABA signaling may contribute to the observed susceptibility in NIL-Ol-1 as ABA signaling is also induced in the susceptible MM in response to PM infection. The restoration of the compromised *Ol-1*-conferred resistance in *Ol-1 × not* further supports a role of ABA for the compromised resistance of NIL-Ol-1 under combined stress.

Jasmonic acid signaling is induced in the resistant NIL-ol-2 challenged with PM, and disruption of JA signaling in the *ol-2 × def* cross results in partial breakdown of resistance. *OPR3* silencing in tomato, which diminished OPDA and JA biosynthesis, resulted in reduced callose deposition (Scalschi et al., 2015), suggesting a connection between JA and callose deposition. Therefore, it is conceivable to draw a cause and effect link between JA-deficiency and lower callose deposition, and enhanced PM susceptibility in *ol-2 × def.* The reversal of susceptibility in *ol-2* crosses with JA and ABA mutants under combined stress, suggests that abiotic stress may act synergistically with the mechanisms contributing to *ol-2*-mediated resistance.

### Performance Costs and Benefits of *epi* and *not* Mutations in NIL-Ol-1 and NIL-ol-2 under Combined Stress

Despite the positive effect of decreasing senescence under combined stress, ABA deficiency had a severe plant performance cost in terms of fresh weight under salt and combined stress. The ABA pathway appears to underlie the antagonistic effects between abiotic and biotic stress (Yasuda et al., 2008; Sanchez-Vallet et al., 2012). Therefore ABA signaling should be studied in more details under combined stress, and include examination of downstream signaling components that enhance disease resistance without compromising abiotic stress adaptation and *vice versa* (Garcia-Andrade et al., 2011).

Although the *epi* mutant increased PM susceptibility, it resulted in a better growth under salt and combined stress. However, the growth penalty of the *Ol-*gene *× epi* plants under control conditions should be taken into account when considering the *epi* mutation for improving stress tolerance of commercially grown tomato under multiple stress conditions. Nevertheless, adapting ET signaling for improving crop resilience is an interesting strategy, which is supported by several studies identifying a positive contribution of ET signaling components in adaptation to abiotic stress (Cheng et al., 2013; Jiang et al., 2013; Peng et al., 2014).

### ABA, JA, and ET Pathways Have no Influence on the Resistance Mediated by the *Ol-4* Gene

In contrast to *Ol-1* and *ol-2*, the resistance mediated by *Ol-4* was stable under all treatments and with all hormone mutant combinations. *Ol-4* is a homolog of the *Mi-1* gene, encoding an NBS-LRR protein (Seifi et al., 2011). It triggers HR in a single epidermal cell in which fungal growth is stopped completely (Bai et al., 2003; Li et al., 2006). *R*-gene resistance is based on effector-triggered immunity (ETI), which is characterized by compensatory relationships between its different signaling components. The ETI response is stronger and more prolonged than PTI (Tsuda et al., 2009), making it more robust and less prone to negative regulation from environmental or genetic factors. Since resistance conferred by *Ol-4* was not affected by the large genetic perturbations disrupting whole hormonal pathways in the hormonal mutants, it has the potential to be stable in combination with larger changes in hormone signaling pathways conferring abiotic stress tolerance. Whether only *Ol-4* has this potential or it is applicable to *R*-genes in general remains to be established.

## Conclusion

Ethylene appears to be central in the plant responses under combined stress, increasing PM susceptibility but promoting salt tolerance. The role of ABA and JA appears to be more complex, as their effect was dependent on the type of resistance and the co-occurrence of salt stress. ABA deficiency appears to limit senescence symptoms, but with significant trade-off between plant salt tolerance and growth. More detailed studies should be carried out to identify specific components of the ABA signaling pathway with fewer pleiotropic effects that can be more effectively implemented to increase combined stress tolerance in crops. Further research is required to delineate the synergistic and antagonistic relationships between signaling components under combined stress and to implement them in precision breeding approaches. Alternatively, the stacking of robust *R*-genes, like *Ol-4*, with well-established abiotic stress tolerance-conferring genes may provide robust resistance under combined abiotic and biotic stress.

## Author Contributions

CK participated in the design of the study, performed the marker selection, phenotypic, histological, gene expression analyses and data analysis and wrote manuscript. AS and ZY generated the crosses and performed an initial phenotypic characterization. AI and HvdS performed the marker selection and phenotypic characterization. CvdW, RV, CvdL, and YB designed the study, supervised this work and assisted with editing the manuscript. All authors read and approved the final manuscript.

## Conflict of Interest Statement

The authors declare that the research was conducted in the absence of any commercial or financial relationships that could be construed as a potential conflict of interest.

## Footnotes

1 http://solgenomics.net

## Supplementary Material

TABLE S1 **Primers used for expression analyses with qRT-PCR**.

TABLE S2 **Multiple comparisons (protected LSD, *P* ≤ 0.05) of disease index and senescence under powdery mildew (PM) and combined stress (PM+Salt).** Statistically significant differences between genotypes are designated with different letters.

FIGURE S1 **Whole plant phenotypes of NIL-Ol-1 and its crosses with mutants under powdery mildew (PM) and in combination with 50 Mm NaCl**.

FIGURE S2 **Na^+^, Cl^-^, K^+^, SO_4_^2-^, Mg^2+^, and Ca^2+^concentration in MM, NIL-Ol-1, and NIL-ol-2 and their respective crosses with *epi* and *not*.** Treatment and labeling scheme are the same as **Figure 3**. Asterisks denote statistically significant differences (*P* ≤ 0.05) between salinity and PM-combined stress for individual genotypes.

FIGURE S3 **Expression of additional genes-markers of hormonal, abiotic, and biotic stress signaling pathways relative to *EF1a*, which was used as a housekeeping gene.** Treatment and labeling scheme are the same as **Figure 4**.
