# Supplementary material for: Ethylene and Abscisic Acid Signaling Pathways Differentially Influence Tomato Resistance to Combined Powdery Mildew and Salt Stress
Source: Front Plant Sci. 2017 Jan 9;7:2009. doi: 10.3389/fpls.2016.02009 (PMC5220069; doi:10.3389/fpls.2016.02009)
Supplement: TABLE S2 — Multiple comparisons (protected LSD, P ≤ 0.05) of disease index and senescence under powdery mildew (PM) and combined stress (PM+Salt). Statistically significant differences between genotypes are designated with different letters. [file Table_2.DOCX]

| **Genotype** | **Treatment** | **Disease Index** | **Significance** | |
| --- | --- | --- | --- | --- |
| ol-2 | PM | 0 | | a |
| ol-2 | PM+Salt | 0 | | a |
| Ol-4x*def* | PM | 0 | | a |
| Ol-4x*def* | PM+Salt | 0 | | a |
| Ol-4 | PM | 0 | | a |
| Ol-4 | PM+Salt | 0 | | a |
| Ol-4x*epi* | PM | 0 | | a |
| Ol-4x*epi* | PM+Salt | 0 | | a |
| Ol-1x*not* | PM | 0.5 | | b |
| ol-2x*not* | PM+Salt | 0.5 | | b |
| ol-2x*def* | PM+Salt | 0.6 | | b |
| Ol-1x*not* | PM+Salt | 0.7 | | bc |
| ol-2x*epi* | PM | 0.8 | | bc |
| ol-2x*def* | PM | 1 | | cd |
| Ol-1 | PM | 1.2 | | de |
| ol-2x*epi* | PM+Salt | 1.2 | | de |
| Ol-1x*def* | PM | 1.4 | | e |
| ol-2x*not* | PM | 1.5 | | e |
| Ol-1 | PM+Salt | 2.4 | | f |
| Ol-1x*def* | PM+Salt | 2.6 | | f |
| Ol-1x*epi* | PM | 3.2 | | g |
| Ol-1x*epi* | PM+Salt | 4.1 | | h |
| MM | PM | 4.2 | | h |
| MM | PM+Salt | 4.8 | | i |
|  |  |  | |  |
| **Genotype** | **Treatment** | **Senescence Index** | | **Significance** |
| Ol-4x*epi* | PM | 0 | | a |
| Ol-4x*epi* | PM+Salt | 0.2 | | ab |
| Ol-1x*not* | PM | 0.2 | | ab |
| ol-2x*epi* | PM | 0.3 | | ab |
| ol-2x*not* | PM | 0.3 | | ab |
| ol-2 | PM | 0.3 | | ab |
| ol-2 | PM+Salt | 0.3 | | ab |
| Ol-4x*def* | PM | 0.375 | | abc |
| Ol-4x*def* | PM+Salt | 0.375 | | abc |
| Ol-1x*epi* | PM | 0.4 | | abc |
| Ol-4 | PM+Salt | 0.4 | | abc |
| ol-2x*def* | PM | 0.5 | | abcd |
| Ol-4 | PM | 0.5 | | abcd |
| Ol-1x*epi* | PM+Salt | 0.6 | | bcde |
| Ol-1 | PM | 0.7 | | cdef |
| ol-2x*def* | PM+Salt | 0.7 | | cdef |
| MM | PM | 0.8 | | def |
| ol-2x*epi* | PM+Salt | 0.8 | | def |
| ol-2x*not* | PM+Salt | 0.9 | | ef |
| Ol-1x*not* | PM+Salt | 1 | | f |
| Ol-1x*def* | PM | 1.5 | | g |
| Ol-1 | PM+Salt | 2.8 | | h |
| MM | PM+Salt | 3.1 | | hi |
| Ol-1x*def* | PM+Salt | 3.3 | | i |
