# Supplementary figures and images for: Ethylene and Abscisic Acid Signaling Pathways Differentially Influence Tomato Resistance to Combined Powdery Mildew and Salt Stress
Source: Front Plant Sci. 2017 Jan 9;7:2009. doi: 10.3389/fpls.2016.02009 (PMC5220069; doi:10.3389/fpls.2016.02009)

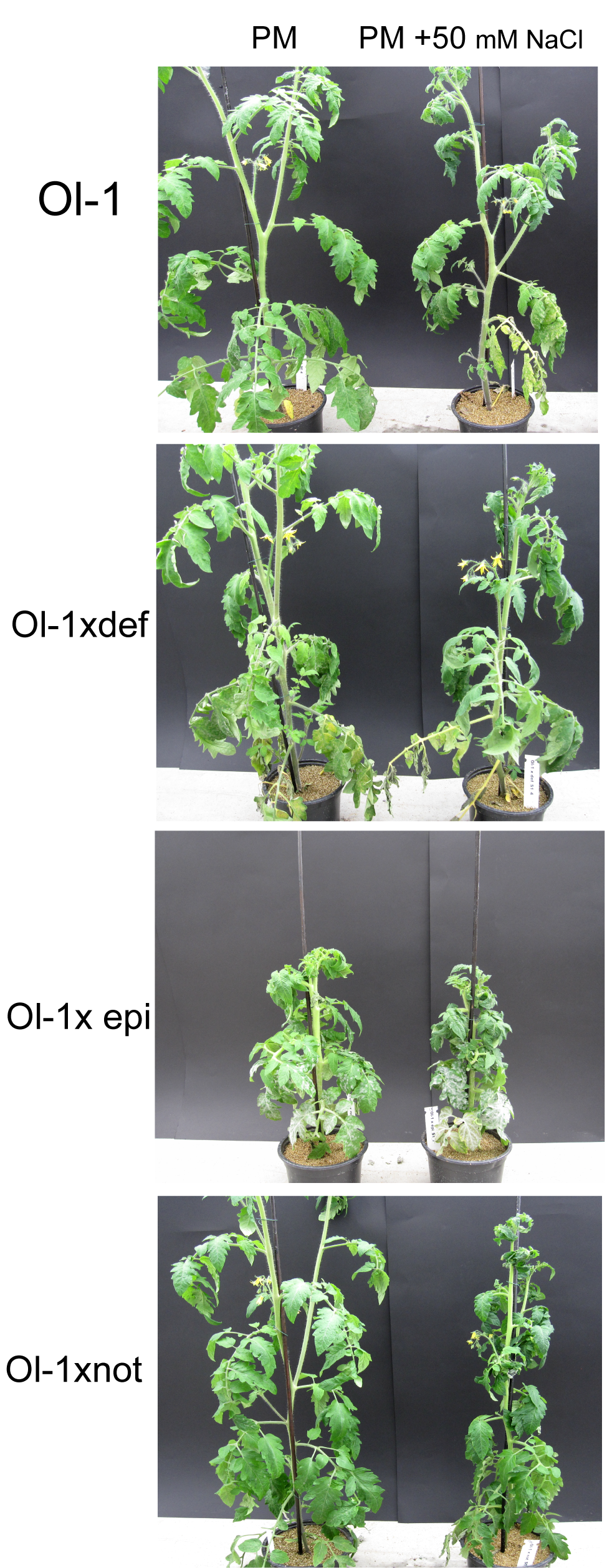

Supplement: FIGURE S1 — Whole plant phenotypes of NIL-Ol-1 and its crosses with mutants under powdery mildew (PM) and in combination with 50 Mm NaCl. [file Image_1.TIF]

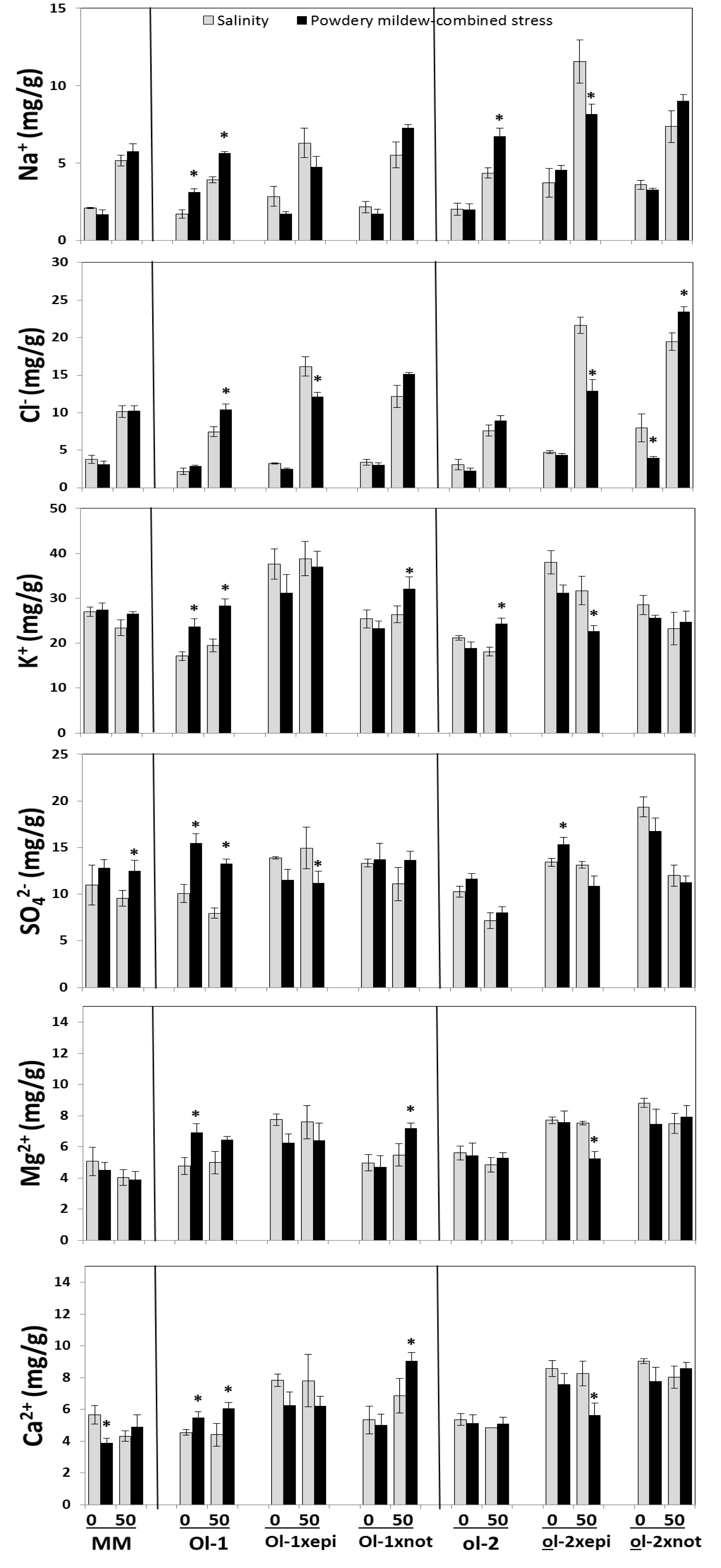

Supplement: FIGURE S2 — Na+, Cl-, K+, SO42-, Mg2+, and Ca2+concentration in MM, NIL-Ol-1, and NIL-ol-2 and their respective crosses with epi and not. Treatment and labeling scheme are the same as Figure 3. Asterisks denote statistically significant differences (P ≤ 0.05) between salinity and PM-combined stress for individual genotypes. [file Image_2.TIF]

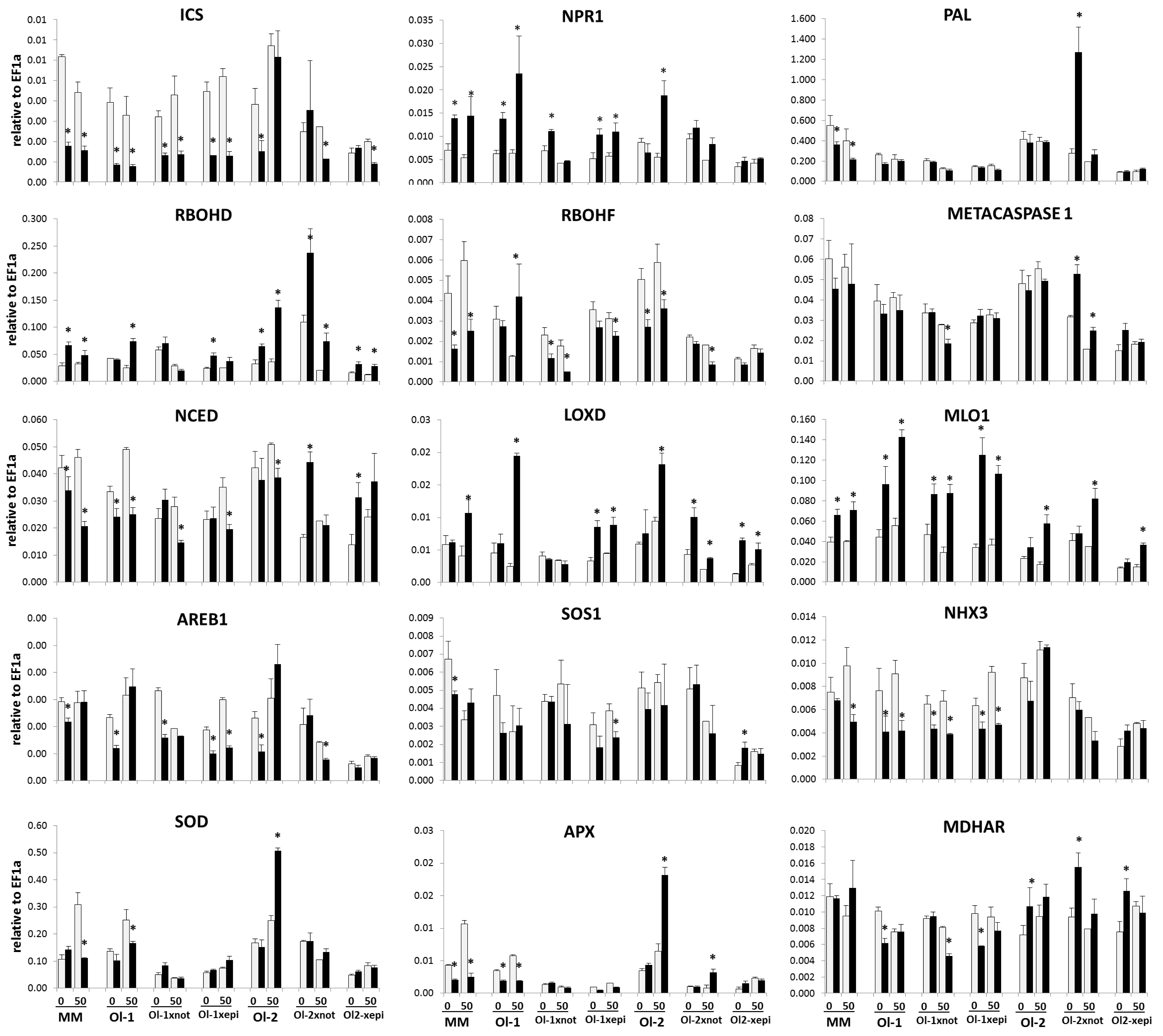

Supplement: FIGURE S3 — Expression of additional genes-markers of hormonal, abiotic, and biotic stress signaling pathways relative to EF1a, which was used as a housekeeping gene. Treatment and labeling scheme are the same as Figure 4. [file Image_3.TIF]
